# Purtscher Retinopathy Following Compressive Chest Trauma: A Case Report

**DOI:** 10.7759/cureus.41100

**Published:** 2023-06-28

**Authors:** Taimoor A Khan, Mohammad A Mehboob, Talha Liaqat, Muhammad A Zahid, Saad H Bhatti

**Affiliations:** 1 Ophthalmology, National University of Medical Sciences, Rawalpindi, PAK; 2 Ophthalmology, Armed Forces Institute of Ophthalmology, Rawalpindi, PAK; 3 Ophthalmology/Vitreoretinal Ophthalmology, Armed Forces Institute of Ophthalmology, Rawalpindi, PAK; 4 Medicine, National University of Medical Sciences, Rawalpindi, PAK; 5 Ophthalmology, Monash Health, Clayton, AUS; 6 Ophthalmology/Corneal and Refractive Surgery, Armed Forces Institute of Ophthalmology, Rawalpindi, PAK

**Keywords:** cotton wool spots, purtscher flecken, hemorrhages, retina, purtscher retinopathy

## Abstract

Purtscher retinopathy (PR) is an occlusive vasculopathy of the retinal microvasculature that classically presents 24-48 hours after compressive chest trauma. Symptoms vary from mild to severe acute visual loss. Characteristic findings on fundus examination such as Purtscher flecken, cotton wool spots, and retinal hemorrhages may also be found. Here, we discuss a case of Purtscher retinopathy due to compressive chest trauma.

## Introduction

Purtscher retinopathy (PR), also known as traumatic retinal angiopathy or lymphorrhagia retinae, is defined as occlusive vasculopathy of the retinal microvasculature associated with retinal hemorrhages, white retinal areas due to microvascular occlusion, and perivascular clearing around the fovea [1].

Omar Purtscher was an Austrian ophthalmologist who described the first known case of Purtscher retinopathy in a middle-aged male who developed vision loss after falling off a tree and developing head trauma. An ocular examination revealed retinal hemorrhages in the posterior poles of both eyes [2]. Omar Purtscher originally proposed that vision loss occurs due to lymph fluid extravasation from retinal vessels due to a sudden increase in vascular pressure due to head trauma. However, since then, multiple other causes have been observed to precipitate clinical features of Purtscher retinopathy, such as pancreatitis, childbirth, HELLP (hemolysis, elevated liver enzymes, and low platelets) syndrome, and renal failure. Thus, the cause of vascular changes may not strictly be related to head trauma but rather some underlying inflammatory changes [3]. Features similar to Purtscher retinopathy can occur in the absence of trauma, and the condition is called Purtscher-like retinopathy.

Patients typically present 24-48 hours after the precipitating event with symptoms of acute loss of vision that ranges from minimal loss to only being able to perceive hand movements. Vision loss may also be associated with vision field defects. Characteristic findings on fundus examination such as Purtscher flecken, cotton wool spots, and retinal hemorrhages may also be found [4].

Here, we discuss a case of Purtscher retinopathy due to compressive chest trauma.

## Case presentation

**Figure 1:**
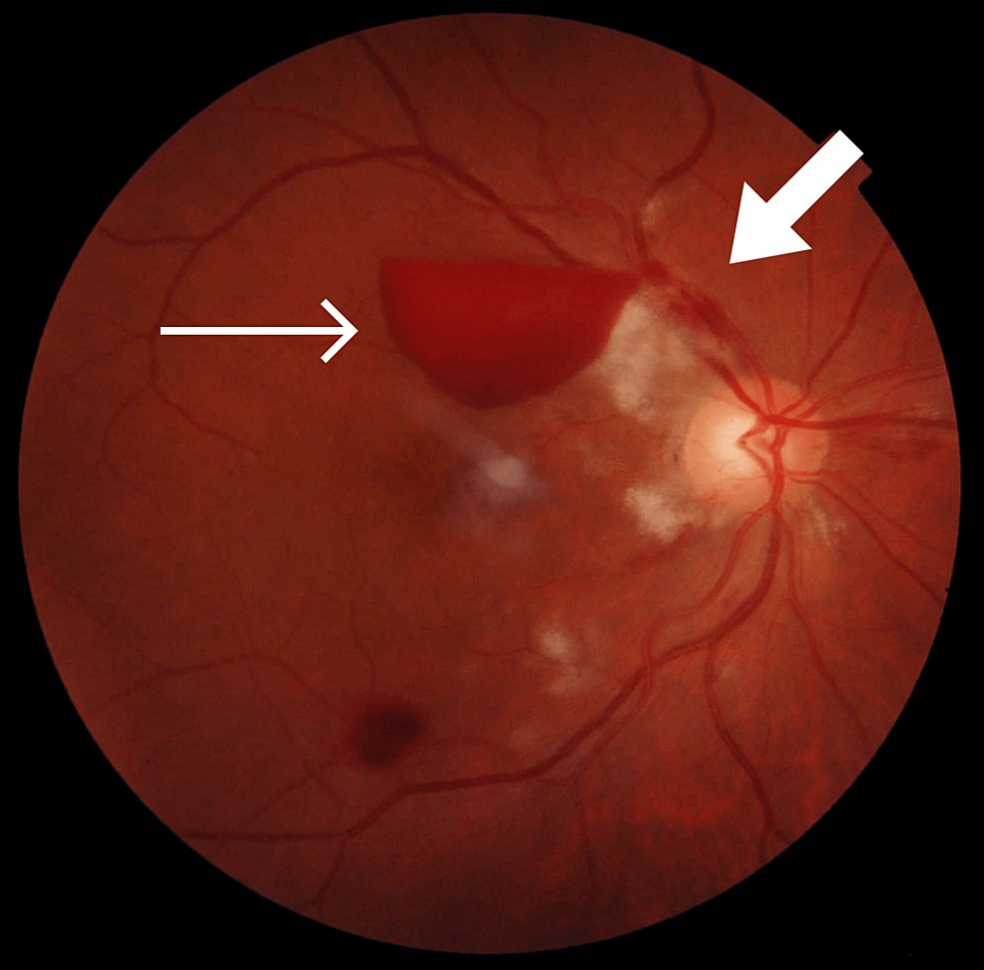
Colored fundus photograph of the right eye showing multiple Purtscher fleckens temporal to the disc (broader arrow), a small superotemporal flame-shaped hemorrhage, a large boat-shaped subhyaloid hemorrhage superior to the fovea (thin arrow), and a small intraretinal hemorrhage along the inferotemporal arcade.

**Figure 2:**
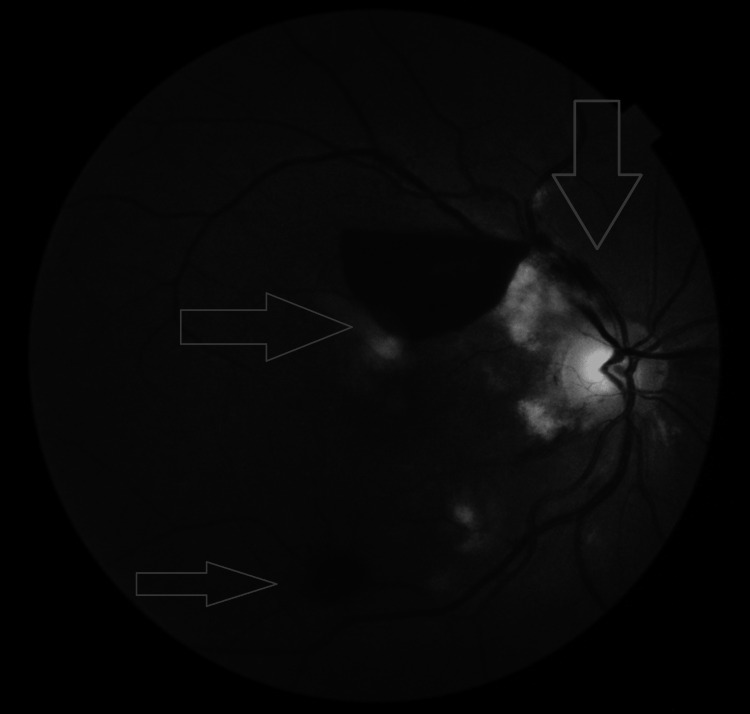
Red-free fundus photograph of the right eye showing multiple hyperintense Purtscher fleckens temporal to the disc, a hypointense small superotemporal flame-shaped hemorrhage, a hypointense large boat-shaped subhyaloid hemorrhage superior to the fovea, and a hypointense small intraretinal hemorrhage along the inferotemporal arcade.

A call for ophthalmic consultation was received for a 35-year-old male who was admitted to the surgical intensive care unit (ICU) on account of pneumothorax due to a fracture of the third and fourth rib secondary to compressive chest trauma in a road traffic accident (RTA). He now complained of sudden persistent painless loss of central vision in the right eye for 24 hours. His visual loss was severe, sudden in onset, and not associated with any pain, redness, floaters, or flashes of light. He has been using glasses for the past 20 years. He denies any significant medical or surgical history and has no known drug allergies. On examination, visual acuity in his right eye was hand movement improving neither by pinhole nor refraction. His visual acuity in the left eye was 6/18, best corrected to 6/6 with -1.00 dioptric sphere refraction. He had +4 relative afferent pupillary defect (RAPD) on the right side. The rest of his anterior segment examination was unremarkable in both eyes. His posterior segment examination revealed a clear vitreous, multiple confluent Purtscher fleckens temporal to the disc extending up to 3 disc diameter, obscuring the underlying retinal vasculature view. There was a small flame-shaped hemorrhage and boat-shaped subhyaloid hemorrhage superior to the fovea along the junction of the medial third and lateral two-thirds of the superotemporal arcade, approximately 2 disc diameter in size. Another intraretinal hemorrhage of approximately two-thirds disc diameter was present on the macula along the junction of the lateral third and medial two-thirds of the inferotemporal arcade (Figures 1, 2).

**Figure 3:**
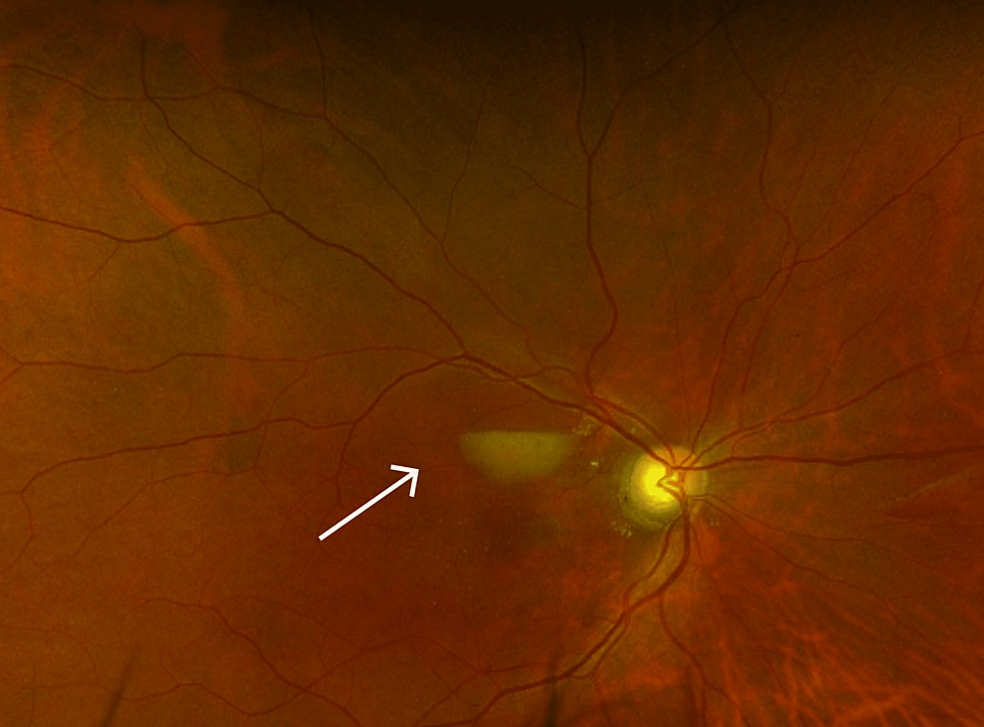
Ultra-wide field colored fundus photograph of the right eye showing resolution of the subhyaloid hemorrhage (white arrow) and Purtscher fleckens.

**Figure 4:**
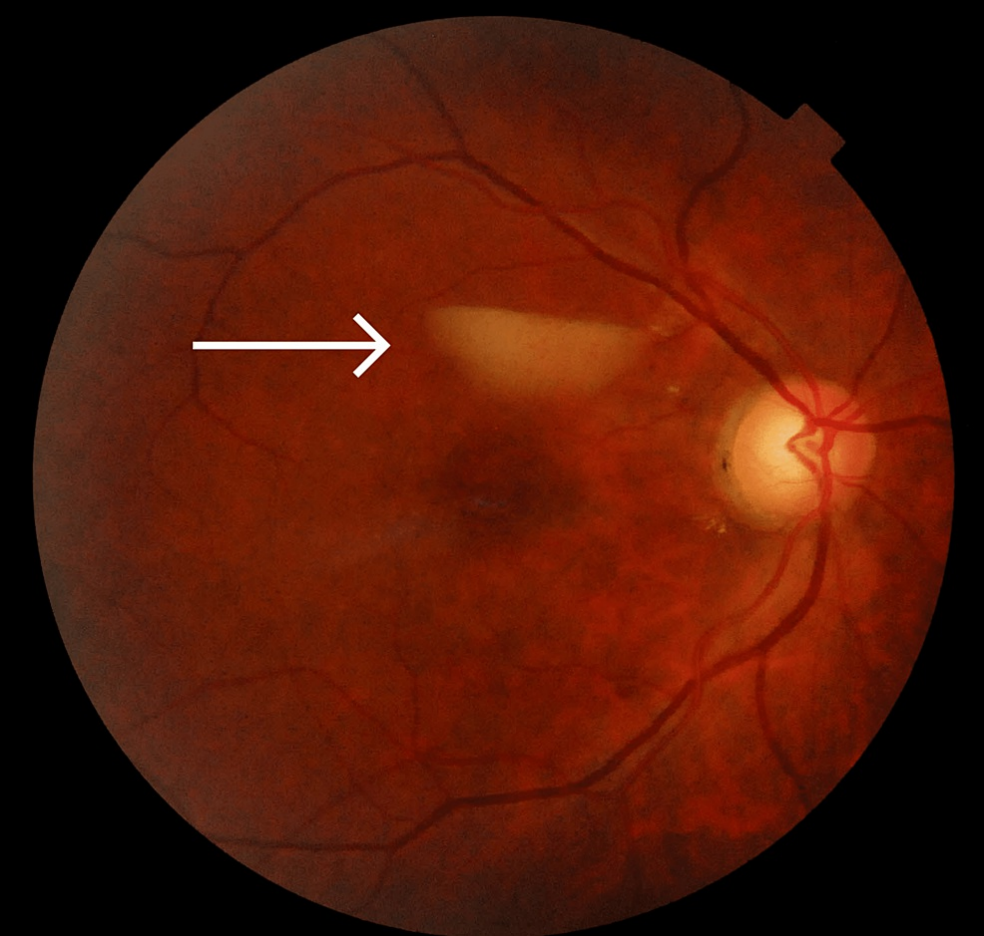
Color fundus photograph of the right eye. The white arrow mark shows resolution of the subhyaloid hemorrhage.

The optic disc had clear margins with a pink neuro-retinal rim, with a cup-to-disc ratio of 0.35, and spontaneous venous pulsations were appreciable. Posterior segment examination of the left eye showed few peripapillary Purtscher fleckens. His optical coherence tomography (OCT) by Spectralis revealed a sub-internal limiting membrane hemorrhage in the right eye, while no subretinal fluid or intraretinal edema in the right or left eye. Computed tomography of the orbit and brain (thin slices) was ordered to rule out a fracture of the optic nerve canal/damage to the visual pathway, which yielded an essentially unremarkable study. Based on the history, clinical examination, and supporting investigations, a diagnosis of Purtscher retinopathy was established. He was planned on a three-day follow-up, initially followed by a weekly follow-up on topical nepafenac 1% eye drops one drop twice daily for the right eye. At four weeks follow-up, there was a subtle improvement in vision to counting fingers at 2 meters. His visual acuity further improved to 6/20 with refraction (-1.25 dioptric sphere) at 10 weeks follow-up with a marked resolution of Purtscher fleckens. Subhyaloid hemorrhage was resolving on 10 weeks follow-up, while the optic nerve showed mild temporal pallor (Figures 3, 4).

## Discussion

Purtscher retinopathy is an occlusive microvasculopathy associated with some form of trauma such as head trauma or chest trauma during chest compressions. Purtscher-like retinopathy is a condition similar to Purtscher retinopathy with identical clinical presentation in the absence of trauma. The precipitating event is some form of an inflammatory condition such as pancreatitis or HELLP syndrome [5]. According to a study conducted in 2007 to determine the incidence and presentation of Purtscher retinopathy, the annual incidence was determined to be 0.24 cases per million population. However, according to the authors of the study, this represents a minimum incidence as the variation in clinical presentation combined with the rarity of the disease and minimal clinical suspicion may lead to underreporting. Additionally, while the vascular changes associated with Purtscher retinopathy may be found in more patients as reported above, symptomatic Purtscher is rare, and thus, the actual incidence is harder to determine [5].

The diagnosis of Purtscher retinopathy is primarily clinical. The most common presenting symptom is acute vision loss. The most characteristic finding is Purtscher flecken, defined as discrete areas of retinal whitening in the retina. The flecken are polygonal and can be of variable sizes. The flecken are found in the areas between the retinal arterioles and venules. Pseudo-cherry red spots and retinal hemorrhages may also be seen. All these findings are confined to the posterior pole of the retina primarily within the macula and the retinal region nasal to the optic disc. Occasionally, other findings such as macular edema and optic disc swelling may also coexist [4,6].

Initially, mechanical trauma was believed to be the primary cause of vasculopathy. However, since then, PR has been seen to occur with different non-traumatic conditions, and so, while trauma may be one of the causes, it is not the only cause and thus not a sufficient explanation behind the pathogenesis of PR. Additionally, if trauma alone caused PR, it should be reflected in terms of the clinical presentation of PR with road traffic accidents (RTAs). However, the incidence of PR is far less than the incidence of RTAs. One theory that proposes a more complete explanation of PR pathogenesis is the microcirculation theory. It proposes retinal microvascular occlusion to be the cause of PR. Severe trauma and inflammatory conditions can cause complement system activation and C5a-induced leukocyte aggregation. Increased inflammation can in turn lead to the activation of platelet aggregation, eventually resulting in small clot formation and microvascular embolization [7].

The prognosis of PR is usually good with most patients reported to recover visual acuity without any specific intervention. This usually occurs within the first week of the development of ocular symptoms. There are no specific interventions or treatment guidelines for PR or Purtscher-like retinopathy. Most patients completely recover within 1-3 months. Glucocorticoid therapy has been used according to a systematic review conducted in China, but it did not reveal any significant differences compared to patients not receiving any treatment [8]. Treatment should primarily be focused on removing the precipitating factors such as underlying pancreatitis or management of acute trauma.

## Conclusions

Purtscher retinopathy is a rare ocular condition that can cause significant anxiety in patients and an alarming clinical presentation. However, the condition itself is fairly benign as long as the underlying cause is managed adequately as evident in our patient who is on his road to a smooth recovery from a very severe visual loss. Keeping it as a potential diagnosis in a case of acute vision loss after any traumatic or acute inflammatory condition can help guide management and provide reassurance to the patient.
